# Evaluation of duplicated reference genes for quantitative real-time PCR analysis in genome unknown hexaploid oat (*Avena sativa* L.)

**DOI:** 10.1186/s13007-020-00679-1

**Published:** 2020-10-15

**Authors:** Zheng Yang, Kai Wang, Usman Aziz, Cuizhu Zhao, Meng Zhang

**Affiliations:** College of Agronomy, Northwest A&F University, Yangling, 712100 Shaanxi China

**Keywords:** Oat, Reference gene, qPCR, Duplicated gene, Polyploid, Gene expression, Endosperm

## Abstract

**Background:**

Oat (*Avena sativa* L.), a hexaploid crop with unknown genome, has valuable nutritional, medicinal and pharmaceutical uses. However, no suitable RGs (reference genes) for qPCR (quantitative real-time PCR) has been documented for oat yet. Single-copy gene is often selected as RG, which is challengeable or impactable in unexplored polyploids.

**Results:**

In this study, eleven candidate RGs, including four duplicated genes, were selected from oat transcriptome. The stability and the optimal combination of these candidate RGs were assessed in 18 oat samples by using four statistical algorithms including the ΔCt method, geNorm, NormFinder and BestKeeper. The most stable RGs for “all samples”, “shoots and roots of seedlings”, “developing seeds” and “developing endosperms” were *EIF4A* (*Eukaryotic initiation factor 4A-3*), *UBC21* (*Ubiquitin-Conjugating Enzyme 21*), *EP* (*Expressed protein*) and *EIF4A* respectively. Among these RGs, *UBC21* was a four-copy duplicated gene. The reliability was validated by the expression patterns of four various genes normalized to the most and the least stable RGs in different sample sets.

**Conclusions:**

Results provide a proof of concept that the duplicated RG is feasible for qPCR in polyploids. To our knowledge, this study is the first systematic research on the optimal RGs for accurate qPCR normalization of gene expression in different organs and tissues of oat.

## Background

Oat (*Avena sativa* L.) is an allohexaploid (2n = 6 ×  = 42) cereal crop with estimated 13 Gb genome [1]. With an upswing in food and industrial utilization, oats are now cultivated worldwide and form an important dietary staple on a global scale [2, 3]. As a wholegrain product, rolled oats are a rich source of minerals, starch and lipids, and they are a predominant supply of soluble fiber β-glucan [4, 5]. Particularly, unlike other cereals, most of the lipids in oat seeds are deposited in cells of oat endosperms which also accumulate starch [6, 7]. Due to the rich constituents, oats also possess different pharmacological purposes like antioxidative, immunomodulatory, antidiabetic and anti-cholesterolaemic effects [8, 9]. Additionally, oat plants are more adapted to severe weather compared to other monocot crops, and they comparatively require fewer pesticide and fertilizers than other food cereals [10, 11]. These features boost oat as an eco-friendly crop with valuable nutrition and pharmaceutical applications. Many classic breeding approaches are already underway to explore and improve oats [12]. Moreover, with the combined advances in molecular biological research and omics technologies, there has been an increasing number of oat studies that focuses on specific genes in molecular breeding endeavors [13, 14].

Gene expression analysis is becoming increasingly important for exploring functions of candidate genes in biological research. Because gene expression is mainly regulated at the transcription level, studies of it are often carried out at the level of mRNA. Techniques for measuring gene expressions commonly include Northern blot, in situ hybridization, semiquantitative reverse transcription PCR, reverse transcription-PCR, microarray and RNA-seq. Among them, quantitative real-rime polymerase chain reaction (qPCR) is more commonly used for measuring mRNA levels of specific genes for its specificity, sensitivity, flexibility, scalability, and most importantly its potential for high throughput [15, 16]. The fluorescent reporter molecules are used in qPCR to monitor the amplification production during each cycle of the PCR reaction. The amounts of qPCR products are generally calculated by the relative quantification compared with stably expressed genes, which is the most robust and straightforward method of accurately quantifying subtle changes [17]. Reference gene (RG) is the prerequisite for gene expression normalization in relative quantification analysis. An unsuitable RG in gene expression assays usually leads to confounding results [18]. Therefore, the validity of an RG is critical for generating reliable and accurate qPCR results [19, 20].

Some housekeeping genes, such as *glyceraldehyde-3-phosphate dehydrogenase*, *beta-actin*, *18S ribosomal RNA*, *elongation factor-1 alpha* and *ubiquitin*, are generally selected as RGs [21, 22]. Nevertheless, previous studies pointed out that the commonly used housekeeping genes might not be suitable for all materials under different experimental conditions [23, 24]. Accordingly, an increasing number of studies have been conducted to identify reliable RGs for various plant materials at different developmental stages. Meanwhile, several statistical algorithms such as geNorm [25], NormFinder [26] and BestKeeper [27], have been developed for the evaluation of RGs for qPCR analysis.

To our best knowledge, RG selection and evaluation in oat have not been reported. Especially, as an allohexaploid crop similar to wheat, oat may mainly contain duplicated genes, and each copy of these duplicated genes may not uniformly express in different samples, which makes it complicated to search proper RGs or design optimal primers [28, 29]. Polyploids such as tobacco, potato, rapeseed, camelina and wheat are widely cultivated and economically important. Single-copy genes are usually used as RGs, although they only account for a small proportion in the genomes of polyploids [30, 31]. In fact, it is worth noting that most researches on RG selection in polyploids neither display nor discuss the copy number of candidate RGs [32–36]. However, with the widespread of omics technology, some of these “single-copy” RGs were proven to be duplicated genes. Moreover, gene duplication cannot be simply determined in a polyploid without sequenced genome, such as oat. Therefore, the examination and validation of duplicated RGs are common concerns for researchers who are facing a polyploid with unknown genome. Taken together, it is indispensable to identify and verify appropriate RGs in oat, and it is also worth evaluating duplicated RGs in such genome unknown species.

In this study, eleven candidate RGs with one or more copies were selected from the transcriptome of hexaploid oat seeds. Because of the nutritious seeds [4, 5], the unique oily endosperms [6, 7], and the different roles of shoots and roots play in stress tolerance [10], seven stages of developing seeds and corresponding endosperms, as well as shoots and roots from two-leaf and three-leaf stages were collected as oat samples separately. The qPCR assays of these 18 samples were performed with specific primer pairs for the left ten candidate RGs after the evaluation of primers designed for them. And the expression stabilities were evaluated using four statistical algorithms including the ΔCt method, geNorm, Normfinder and BestKeeper. The comprehensive ranking of the optimal RG for each sample sets was generated by geometric means of four rankings. The expression levels of four various genes in different sample sets were normalized to the most and the least stable RGs for verifying the reliability of the evaluation results. The results of this study present a comprehensive screening of RGs in diverse samples of oat for the first time, and furthermore provide a foundation of accurate gene expression analysis for this crop. Moreover, this study also demonstrates the feasible use of duplicated RGs in hexaploid oat, and an effective system dealing with selection of duplicated RGs in polyploids was also discussed.

## Results

### Selection of oat candidate reference genes

Due to the absence of genomic sequencing for oat, the exclusive released transcriptome of oat seeds [1] was used as the BLAST database, and the sequences of up to 19 RGs published in previous articles [37–40] or collected online were used as query data in TBLASTN to search their homologs in oat seeds transcriptome. However, several query sequences were not found with any BLAST hits in that oat transcriptome. Among matched subject transcripts, some of them were too short for qPCR primer designing. Consequently, a total of 11 candidate RGs, namely *Protein Phosphatase 2A Subunit A3* (*PP2A*), *Polyubiquitin 10* (*UBQ10*), *Ubiquitin-Conjugating Enzyme 21* (*UBC21*), *Elongation factor 1-Alpha* (*EF1A*), *Glyceraldehyde-3-phosphate Dehydrogenase C Subunit 1* (*GAPDH1*), *18S ribosomal RNA* (*18S*), *Heterogeneous nuclear ribonucleoprotein 27C* (*HNR*), *Expressed protein* (*EP*), *TBC1 domain family member 22A* (*TBC*), *Tubulin alpha-6 chain* (*TUA6*) and *Eukaryotic initiation factor 4A-3* (*EIF4A*), were identified as candidates for qPCR primer designing and their sequences were listed in Additional file 1. In hexaploid oat, it was not surprising that duplicated genes in its transcriptome was identified. Among 11 candidate RGs, *18S* and *GAPDH1* were matched with two copies, while *TUA6* and *UBC21* had three and four transcripts respectively, and others only had one best BLAST hit (Table 1).

**Table 1:** Candidate reference genes, amplicon characteristics and primer sequences

Gene	Locus name in Oat Transcriptome [1]	RPKM [1]	Primers (5′–3′)	Tm (℃)	Amplicon length	Efficiency (%)	R^2^
*PP2A*	Locus_1956_Transcript_6/10_Confidence_0.500_Length_1648	27.3738	F: GCCCTGAGCCTACAAGAACGG	63.6	196 bp	100.4	1
			R: GCTGAGCGAACATGCTGAGAT	60.3			
*UBQ10*	Locus_4160_Transcript_1/1_Confidence_1.000_Length_1516	12.1493	F: GATCTCAGCTCTAGCGAATCTCC	59.4	136 bp	94.2	1
			R: ATCCCTTCCTTGTCTTGAATCTTG	60.9			
*EF1A*	Locus_565_Transcript_5/10_Confidence_0.529_Length_863	347.2181	F: GTGAAGATGATTCCCACCAAGC	60.9	87 bp	96.0	1
			R: CCTCATGTCACGCACAGCAAA	62.7			
*HNR*	Locus_4951_Transcript_1/4_Confidence_0.667_Length_1455	12.2775	F: ATTGGGTTTGTCACTTTCCGTAG	60.2	134 bp	101.5	1.000
			R: CTTGGAGGGTGTCTCGCATCT	61.4			
*EP*	Locus_827_Transcript_1/2_Confidence_1.000_Length_1359	19.8139	F: GCACAAGTGATGCCAGAATAGC	60.0	193 bp	112.4	1
			R: CGAGATGCATTAGATTCGTTGG	60.1			
*TBC*	Locus_9878_Transcript_1/1_Confidence_1.000_Length_1218	4.3017	F: TCCTCTTTCACCTCCCGATTAC	60.0	98 bp	92.7	1
			R: CAGATGCTTGCCCTTCTACCTC	60.7			
*EIF4A*	Locus_3892_Transcript_3/4_Confidence_0.667_Length_1160	45.0042	F: TCTCGCAGGATACGGATGTCG	63.3	88 bp	100.8	0.99
			R: TCCATCGCATTGGTCGCTCT	63.6			
*18S*	Locus_29_Transcript_3/10_Confidence_0.100_Length_1865	1067.4606	F: TTCTTAGTTGGTGGAGCGATTT	58.7	150 bp	100.4	1
	Locus_29_Transcript_4/10_Confidence_0.100_Length_1865	1052.7564	R: CCTGTTATTGCCTCAAACTTCC	58.5			
*GAPDH1*	Locus_535_Transcript_3/4_Confidence_0.500_Length_1198	291.0028	F: CTTCAACATCATTCCCAGCAG	58.0	288 bp	110.7	1
	Locus_1038_Transcript_1/4_Confidence_0.583_Length_1013	393.7014	R: GCCTTGGCGTCAAAGATGCT	62.6			
*TUA6*	Locus_855_Transcript_1/4_Confidence_0.727_Length_1544	28.0927	F: CCCAACAATGTGAAGTCCAGC	60.2	121 bp	-	-
	Locus_882_Transcript_1/5_Confidence_0.692_Length_1361	164.8515	R: TGAACTGCTCACTCACCCTCC	59.7			
	Locus_3988_Transcript_3/5_Confidence_0.615_Length_1286	24.3489					
*UBC21*	Locus_10069_Transcript_1/3_Confidence_0.750_Length_710	7.3384	F: GCCCATCGGAGACACCTTTTG	64.0	133 bp	97.8	0.99
	Locus_10069_Transcript_2/3_Confidence_0.750_Length_723	6.3587	R: CCTGTCTTGAAGTGAACATTTGG	59.1			
	Locus_3507_Transcript_3/4_Confidence_0.500_Length_967	13.6740					
	Locus_3507_Transcript_2/4_Confidence_0.700_Length_1018	16.8311					

Both the locus name and the RPKM value were obtained from Gutierrez-Gonzalez et al. [1]

Considering the limited information from one set of oat transcriptome data, the genes with only one BLAST hit were insufficient to be considered as single-copy genes. Potential additional copies of them and their expression differentiation among tissues might affect their validity as RGs. Therefore, the genes with multiple hits were also included in the test of their feasibility as RGs. Clearly, it was impractical to design qPCR primers for duplicate genes which share relatively low similarities (i.e. less than 60%). Thus, the sequence similarities of these duplicated genes and their expression levels were characterized first. Sequence alignment showed that two *18S* genes had up to 99.84% similarity in their coding regions, followed by 97% similarity among four *UBC21* genes and 83.33% between two *GAPDH1* genes, and three *TUA6* genes merely displayed approximately 72% similarity. Additionally, the expression level indicated by the RPKM (Reads Per Kilobase per Million mapped reads) value of each transcript from the same homologs could be similar to each other or vary considerably (Table 1). In details, the expression levels of two *18S* genes were almost the same at 1,067 and 1,053. Contrary to *18S* genes, among four *UBC21* genes, two of them showed two times more mRNA accumulation than other two, and the RPKM value of one *TUV6* gene was even seven-fold higher than the other two. These results suggested that duplicated genes could be differentially expressed and one transcript of them might not represent them all at least at the expression level, even only in one specific organ. Therefore, primers for qPCR of these duplicated genes were designed in their identical regions.

### Primer verification and PCR amplification efficiency

**Fig. 1:**
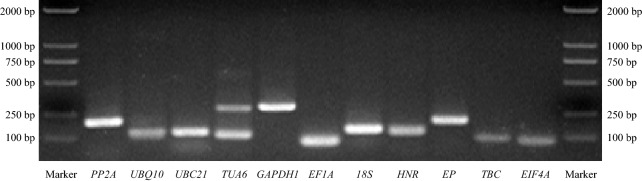
Specificity of primers and amplificon lengths. Specific product lengths of each reference gene were indicated after 1.5% agarose gel electrophoresis. Marker represents Marker DL2000

**Fig. 2:**
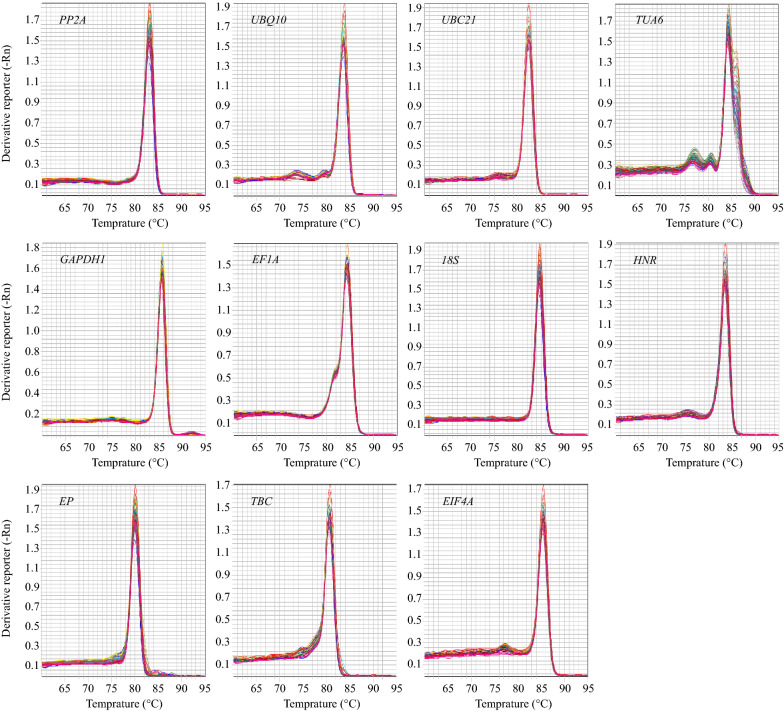
Melt curves of qPCR amplification of 11 candidate reference genes

The primer specificity for candidate RGs was verified by both regular PCR and qPCR, and the cDNA of oat shoots from three-leaf stage seedlings was used as templates. Based on agarose gel electrophoresis, the amplification product sizes ranged from 87 bp of *EF1A* to 288 bp of *GAPDH1* (Fig. 1, Table 1). Specific amplicon was amplified by most pairs of primers, apart from those of *TUA6* (Fig. 1). Similar conclusion was drawn by the number of peaks in melting curve analysis. Only the melt curve of *TUA6* amplicon contained an evident peak noise, which further confirmed the inevitable mispairing of this pair of primers. Meanwhile, other primer pairs produced specific amplificons based on the single peak in their melt curves (Fig. 2). In primers designed for qPCR, over two different mispairing nucleotides on primer can lead to a distinction of two similar sequences [41]. Because there is no other identical region on three *TUA6* transcripts as an alternative priming position, *TUA6* was excluded in following experiments.

The amplification efficiencies of other ten RG primers varied from 92.7% for *TBC* to 112.4% for *EP*, all of which were in the reliable section from 90 to 115% [42]. The correlation coefficient values (R^2^) ranged from 0.993 for *EIF4A* and *UBC21* to 1.000 for *HNR*, which indicated that these primer pairs were highly specific to their targeted region. Other information including the primer sequences and primer characteristics of candidate RGs were all summarized in Table 1.

### Expression stabilities of candidate RGs

To evaluate whether the ten candidate RGs are suitable for qPCR analysis in various organs and tissues of oat, 18 samples were named as four experimental sets: all samples, shoot and root (seedlings), developing seed (seeds) and developing endosperm (endosperms).

**Fig. 3:**
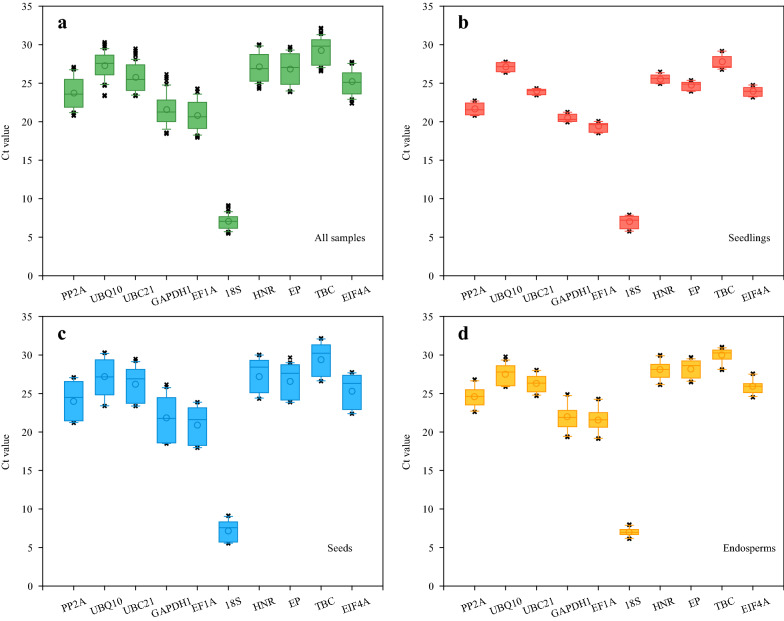
Boxplot analysis of Ct values of ten candidate reference genes in all samples (**a**), seedlings including shoots and roots (**b**), developing seeds (**c**) and developing endosperms (**d**). The boxes indicates the 25th and 75th percentiles. The line across the box represents the median. The circle in the middle of the box shows the mean value. The whisker shows the maximum and minimum values, respectively. × represents the extremum values

The qPCR results were firstly displayed using Ct values in boxplot analysis (Fig. 3) and then evaluated by algorithms including the ΔCt method, geNorm, NormFinder and BestKeeper (Table 2). Corresponding index values for determining gene expression stability were listed in the brackets of each RG (Table 2). The lower these index values are, the higher the gene expression stabilities are. In all sample set, the Ct values ranged from 7.05 of *18S* to 29.23 of *TBC* (Fig. 3a). The *18S* displayed the highest expression and the least variation, whereas *GADPH1* showed the least stability (Table 2). The geNorm and NormFinder analyses both exhibited that *HNR* and *EIF4A* were the top two stable RGs while *18S* was the least. But similar to the results of the ΔCt method, *18S* ranked the first in the BestKeeper analysis.

**Table 2:** Expression stability rankings of 10 candidate RGs using the ΔCt method, geNorm, NormFinder and BestKeeper

Ranking numbers		1	2	3	4	5	6	7	8	9	10
All samples	ΔCt	*18S* (0.95)	*EIF4A* (1.67)	*TBC* (1.71)	*UBQ10* (1.74)	*UBC21* (1.82)	*HNR* (1.85)	*EF1A* (1.94)	*EP* (2.02)	*PP2A* (2.05)	*GAPDH1* (2.06)
	geNorm	*HNR* (0.68)	*EIF4A* (0.70)	*EF1A* (0.71)	*PP2A* (0.78)	*UBC21* (0.78)	*TBC* (0.83)	*EP* (0.92)	*GAPDH1* (0.93)	*UBQ10* (1.09)	*18S* (1.33)
	NormFinder	*EIF4A* (0.15)	*HNR* (0.21)	*EF1A* (0.24)	*UBC21* (0.34)	*PP2A* (0.35)	*TBC* (0.38)	*GAPDH1* (0.49)	*EP* (0.51)	*UBQ10* (0.62)	*18S* (0.85)
	BestKeeper	*18S* (0.78)	*EIF4A* (1.42)	*UBQ10* (1.43)	*TBC* (1.51)	*HNR* (1.68)	*GAPDH1* (1.68)	*UBC21* (1.69)	*EF1A* (1.74)	*EP* (1.80)	*PP2A* (1.80)
Seedlings	ΔCt	*UBC21* (0.34)	*GAPDH1* (0.50)	*EP* (0.51)	*HNR* (0.56)	*EF1A* (0.57)	*UBQ10* (0.60)	*EIF4A* (0.60)	*PP2A* (0.73)	*18S* (0.80)	*TBC* (0.91)
	geNorm	*EIF4A* (0.39)	*HNR* (0.42)	*EP* (0.44)	*UBQ10* (0.45)	*PP2A* (0.45)	*EF1A* (0.50)	*UBC21* (0.51)	*TBC* (0.65)	*18S* (0.76)	*GAPDH1* (0.85)
	NormFinder	*HNR* (0.13)	*PP2A* (0.14)	*UBC21* (0.18)	*EIF4A* (0.21)	*EP* (0.29)	*UBQ10* (0.35)	*EF1A* (0.35)	*TBC* (0.40)	*GAPDH1* (0.42)	*18S* (0.63)
	BestKeeper	*UBC21* (0.25)	*EP* (0.38)	*GAPDH1* (0.44)	*EF1A* (0.45)	*HNR* (0.48)	*EIF4A* (0.55)	*UBQ10* (0.57)	*PP2A* (0.65)	*18S* (0.66)	*TBC* (0.85)
Seeds	ΔCt	*18S* (1.32)	*TBC* (2.12)	*EIF4A* (2.16)	*EP* (2.23)	*HNR* (2.26)	*UBC21* (2.34)	*PP2A* (2.37)	*EF1A* (2.39)	*UBQ10* (2.46)	*GAPDH1* (2.83)
	geNorm	*EF1A* (0.56)	*EP* (0.56)	*HNR* (0.58)	*PP2A* (0.61)	*EIF4A* (0.61)	*UBC21* (0.71)	*TBC* (0.83)	*UBQ10* (0.86)	*GAPDH1* (0.90)	*18S* (1.18)
	NormFinder	*EF1A* (0.11)	*EP* (0.12)	*HNR* (0.16)	*PP2A* (0.19)	*EIF4A* (0.22)	*UBC21* (0.33)	*TBC* (0.46)	*UBQ10* (0.48)	*GAPDH1* (0.55)	*18S* (0.76)
	BestKeeper	*18S* (1.19)	*EIF4A (1.99)*	*TBC* (2.00)	*EP*(2.03)	*HNR* (2.06)	*UBQ10* (2.13)	*PP2A* (2.18)	*UBC21* (2.20)	*EF1A* (2.24)	*GAPDH1* (2.45)
Endosperms	ΔCt	*18S* (0.54)	*EIF4A* (0.87)	*TBC* (0.92)	*UBC21* (1.08)	*EP* (1.13)	*HNR* (1.13)	*UBQ10* (1.34)	*PP2A* (1.36)	*EF1A* (1.52)	*GAPDH1* (1.63)
	geNorm	*HNR* (0.51)	*PP2A* (0.52)	*UBC21* (0.55)	*EIF4A* (0.57)	*EP* (0.63)	*EF1A* (0.64)	*UBQ10* (0.68)	*GAPDH1* (0.70)	*TBC* (0.72)	*18S* (0.98)
	NormFinder	*EIF4A* (0.06)	*HNR* (0.06)	*UBQ10* (0.14)	*EP* (0.14)	*PP2A* (0.16)	*EF1A* (0.24)	*UBC21* (0.24)	*TBC* (0.40)	*18S* (0.48)	*GAPDH1* (0.57)
	BestKeeper	*18S* (0.41)	*EIF4A* (0.65)	*TBC* (0.79)	*UBC21* (0.89)	*HNR* (0.90)	*EP* (1.03)	*PP2A* (1.05)	*EF1A* (1.18)	*UBQ10* (1.20)	*GAPDH1* (1.24)

Values in brackets are STDEV for ΔCt, M values for geNorm, SVs (stability values) for NormFinder and CV ± SD values for BestKeeper (*CV* the coefficient of variance, *SD* standard deviation)

In seedling set, the STDEV values of all candidate RGs were obviously lower than any other sample sets (Fig. 3b; Table 2). The Ct values varied from 7.01 of *18S* to 27.76 of *TBC*. Based on the ΔCt method and the BestKeeper analysis, *UBC21* had the most stable expression, followed by *GAPDH1* and *EP*. However, *GAPDH1* and *18S* were the least stably expressed RGs calculated by geNorm and NormFinder. Furthermore, *HNR* performed relatively better than most of RGs in all four algorithms.

In seed set, candidate RGs displayed the most variation and the least stability among four experimental sets (Fig. 3c; Table 2), which indicated the complicated regulation network of gene expressions during developing oat seeds. *18S* still had the minimum Ct value of 7.13, which indicated its highest expression level among different stages of oat seeds and accorded with the highest RPKM values in oat seed transcriptome (Table 1). *GAPDH1* ranked last in the ΔCt method and BestKeeper analysis, and ranked the second last in geNorm and NormFinder, respectively. Additionally, the rankings of candidate RGs in geNorm and NormFinder were exactly the same, among which *EF1A* and *EP* were the most two stable RGs.

In endosperm set, the distribution of Ct values was similar to that of oat seeds, but the variations and stabilities were distinctly different between them (Fig. 3d; Table 2). Similar conclusions were drawn by the ΔCt method and the BestKeeper analyses, that *18S* and *EIF4A* were the most reliable RGs while *GAPDH1* was the least. In the geNorm analysis, though *HNR* was the greatest RG, the M value of it was very close to that of *PP2A*, *UBC21* and *EIF4A*.

**Fig. 4:**
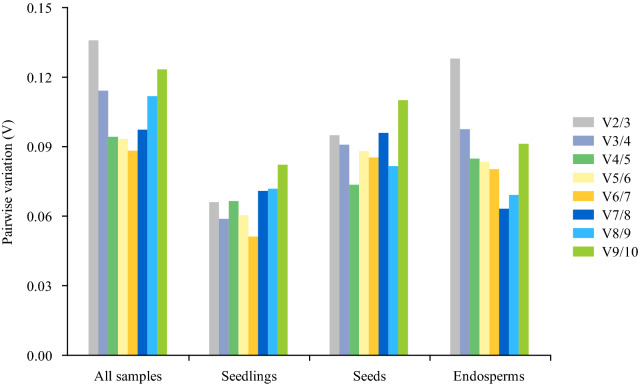
Pairwise variation (Vn/Vn + 1) analysis of the optimal number among ten candidate reference genes in different experimental sets

Previous research had proven that the conventional use of a single RG for normalization led to relatively large errors in qPCR results [25]. The pairwise variation analysis provided an accurate standard to select the minimum number of RGs by comparing the Vn/Vn + 1 values with 0.15. Once the Vn/Vn + 1 value was lower than 0.15, the top n RGs should be combined as the internal standard gene set. As shown in Fig. 4, the V2/V3 values of four experimental sets were all less than 0.15, which indicated that the two RG combinations were reliable enough for result normalization in them. According to this conclusion, the geometric means of all candidate RGs’ ranking values given by four algorithms were calculated, and the best two RGs for each sample set were listed in Table 3. Meanwhile, the least stable RGs of four sample sets were also displayed. To be precise, *EIF4A* + *HNR* was the most stable RG set across all samples and for developing endosperms. The best RG set for seedling samples was *UBC21* + *HNR*, and the optimal RG set for developing seeds was *EP* + *EF1A*. However, *GAPDH1* was the least recommended RG for both seeds and endosperms, and *18S* was particularly unstable for shoots and roots of seedling.

**Table 3:** Comprehensive stability rankings of 10 candidate RGs

All samples		Seedlings		Seeds		Endosperms	
Most	Least	Most	Least	Most	Least	Most	Least
*EIF4A*	*EP*	*UBC21*	*18S*	*EP*	*GAPDH1*	*EIF4A*	*GAPDH1*
*HNR*		*HNR*		*EF1A*		*HNR*	

Ranking values are geometric means of ranking values from the ΔCt method, geNorm, NormFinder and BestKeeper

### Validation of candidate reference genes

**Fig. 5:**
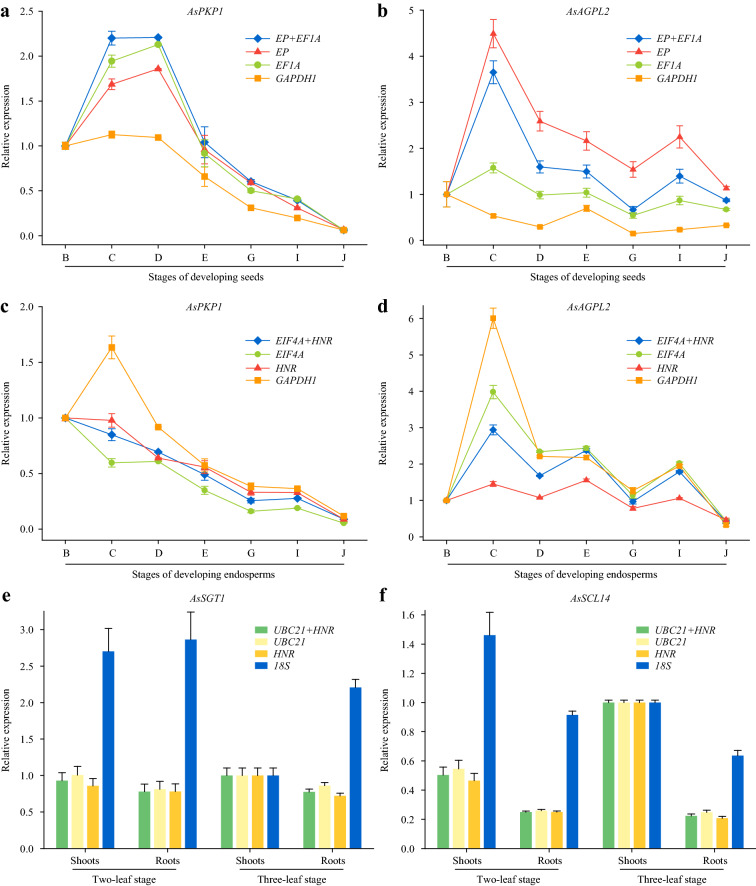
Relative expression level of various genes in different sample sets using the most and the least stable reference genes for normalization. **a**, **b** Relative expression levels of *AsPKP1* (**a**) and *AsAGPL2* (**b**) in developing seeds. *EP+EF1A* represents the most stable RG set and *GAPDH1* is the least stable RG. Error bars indicate SD of three replicates. The B, C, D, E, G, I and J refer to developing stages of oat seeds [7]. **c**, **d** Relative expression levels of *AsPKP1* (**c**) and *AsAGPL2* (**d**) in developing endosperms. *EIF4A+HNR* represents the most stable RG set and *GAPDH1* is the least stable RG. Error bars indicate SD of three replicates. The B, C, D, E, G, I and J refer to developing stages of oat seeds [7]. **e**, **f** Relative expression levels of *AsSGT1* (**e**) and *AsSCL14* (**f**) in shoots and roots of seedlings at two-leaf and three-leaf stage. *UBC21+HNR* represents the most stable RG set and *18S* is the least stable RG. Error bars indicate SD of three replicates

*PKP1* (*Plastidial Pyruvate Kinase 1*) and *AGPL2* (*ADP-glucose pyrophosphorylase large subunit 2*) play important roles in glycolysis [43] and starch biosynthesis [44], respectively. Homologs of *SGT1* [*suppressor of the G2 allele of SKP1* (S-phase kinase-associated protein 1)] and *SCL14* (S*carecrow-Like 14*) in wheat were reported in wheat seedling growth studies [45, 46]. To confirm the reliability of the selected best RG sets after the comprehensive analysis above, the expression levels of *AsPKP1* and *AsAGPL2* were detected in developing seeds and developing endosperms, while those of *AsSGT1* and *AsSCL14* were detected in shoots and roots of seedlings, respectively. In the meantime, the RG with the comprehensively lowest stability in each sample set was used for gene expression normalization as a negative control (Table 3; Fig. 5).

In developing oat seeds, it was evident that the expression patterns of *AsPKP1* and *AsAGPL2* normalized by *EP* + *EF1A*, *EP* and *EF1A* were mainly similar, and were obviously different from that by *GAPDH1* (Fig. 5a, b). When the *EP* + *EF1A* set was used for normalization, the relative expression of *AsPKP1* was 2.2 times higher at stage C than that of stage B, and increase slightly at stage D subsequently, followed by continuous decrease till stage J. However, almost no difference was shown in the expression trend of *GAPDH1* normalization results from stage B to D (Fig. 5a). The expression level of *AsGPL2* normalized by *EP*, *EF1A* and their combination showed an evident increase from stage B to C, while that normalized by *GAPDH1* kept decreasing in same developing period (Fig. 5b). Similar differences were also found in developing endosperms (Fig. 5c, d). When the most stable RG set “*EIF4A* + *HNR*” was used for normalization together and separately, the transcript abundance of *AsPKP1* dropped steadily during the whole developmental stages of endosperms. Conversely, the relative expression of *AsPKP1* showed a sharp increase at stage C compared to that of stage B when *GAPDH1* was used for normalization (Fig. 5c). The expression level of *AsGPL2* varied much more when normalized by *GAPDH1* compared with that normalized by *EIF4A* and *HNR* together or separately (Fig. 5d).

In two-leaf and three-leaf stages of oat seedlings, the relative expressions of *AsSGT1* and *AsSCL14* normalized by optimal *UBC21*, *HNR* and their combination showed almost the same levels, which were evidently different from those normalized by the least stable RG *18S* (Fig. 5e, f). When *UBC21* and *HNR* were used for normalization, *AsSGT1* in seedling roots always showed tiny decreases compared with that in shoots at two different stages. However, the relative expression level of *AsSGT1* even displayed twofold more change between shoots and roots at three-leaf stage, when *18S* was used as RG (Fig. 5e). Thus, these results confirmed the validation and the reliability of the identified RG sets.

## Discussion

qPCR is a powerful tool for analyzing gene expression, with good specificity, high accuracy, great efficiency, and excellent reproducibility. However, numerous studies have shown that the reference genes, such as *GAPDH* and *Actin*, which are used to normalize the data in qPCR studies, may not remain stable across all kinds of samples [47, 48]. In this study, RGs for various organs and tissues of oat were selected and evaluated systematically for the first time.

All candidate RGs in this study were frequently used housekeeping genes. Practically, other functional genes with relatively stable expression levels among different samples were also reported as candidate RGs, for example, a *Dual Specificity Protein Phosphatase* in *Setaria viridis* [24] and a *s-Adenosyl methionine decarboxylase* in *Eriobotrya japonica* [49]. However, the only released oat transcriptome provided data derived from pooled samples of four developmental stages [1], which also limits an exploration for new RGs *in silicon*.

Qualified RGs are suggested to have a low and consistent copy number in different varieties of the same species [50]. Polyploid crops are commonly cultured in agriculture and they normally have amount of multi-copy genes. In *Brassica napus*, a typical tetraploid cash crop, only 9.0% of genes own less than 2 copies (one copy: 3.98%; two copies: 5.02%) and up to 71.53% genes even have over six copies according to the genome-wide analysis. To search proper candidate RGs in *Brassica napus*, only genes with one or two copies were taken into consideration and corresponding qPCR primers were designed in the consistent part of multi-copy genes [31]. In wheat, another hexaploid crop closely related to oat, nearly 90% are present in at least three complete copies generated from the duplication in allopolyploidization [51]. But the criteria for choosing RGs in different wheat varieties or under different conditions remain similar. Basically, any candidate genes with more than two isoforms were excluded and the coincident regions among genes with less than two homologs were used for primer designing [30, 52]. In this study, due to the high estimated percentage of duplicated genes in oat, one to four copy numbers of RGs were all considered as candidates, among which *18S*, *GAPDH1*, *TUV6* and *UBC21* are duplicated genes (Table 1). The two-copy *18S* performed well in three sample sets when evaluated by the ΔCt method (Table 2), and the four-copy *UBC21* even ranked as the most stable RG for oat seedlings (Table 3). These results provide a proof of concept that duplicated RG is feasible and valid in polyploid oat. Additionally, though the 72% similarity among three *TUV6* copies was not quite low, it was still difficult to target continuous identical regions for qPCR primer designing. Thus, this study also put up that the primer designing for multi-copy RGs should also be based on the sequence similarity and continuity among all transcripts.

Polyploids are universally widespread and have important functionalities. However, the large proportion of duplicated genes in their genome leads to great difficulty of the unigene assembly and a high cost of the genome sequencing [51, 53]. As a typical example of polyploids without any reference genome, oat only has one released transcriptome of its developing seeds [13]. Theoretically, the copy number of assembled transcripts might not accord with that of duplicated genes in genome. In this study, candidate RGs with one to four copies were evaluated together, but the copy numbers for them require validation once the whole genome of oat gets completed. Besides, different copies of duplicated genes usually display diverse tissue specificity or display various expression levels even in the same organ [54, 55]. As shown in Table 1, different transcripts of *TUV6* and *UBC21* also showed two to seven-fold variation among their RPKM values in oat seed transcriptome. Further considering that these so-called “single-copy” genes were deduced from limited transcriptome data and have not been supported by genome sequences, they might not represent the total expression level of possible duplicated genes. Therefore, selecting duplicated genes and priming in their identical region may help to eliminate the effect of their potential differentiation on expression in different tissues. Meanwhile, to represent the integral expression level of an RG, the primers for qPCR should be designed in identical regions between homologs. In addition, to be clear, the stabilities evaluated in present study specifically referred to primers generated from existing sequences. The applicability of certain RG demands other assessment with more omics data.

As a traditional cereal crop, researches of oat mainly focus on the food science [4]. Scarce molecular biological studies of oat, especially about gene functions, limit the validation of chosen RGs. Among four genes used for validation in this study, only *AsPKP1* was analyzed in oat previously [14]. Alternatively, three more genes well-studied in wheat were selected to provide more evidences. The expression patterns of these four genes normalized by the optimal RGs were highly consistent, and showed obvious difference compared to those normalized by the worst RG (Fig. 5). Besides, the expression pattern of *AsPKP1* was quite similar to that of *PKP1* homologs in other plant seeds, such as Arabidopsis [56]. As a homolog of *AsSGT1* in wheat, the expression level of *TaSGT1* in seedling shoots is also slightly higher than that in roots [45]. It would be more convincing to compare these expression results with transcriptome data. However, the only transcriptome of mixed developing oat seeds [1] restricts this attempt.

Previous studies on RG selection mainly focus on different organs and tissues from the same species or focus on various treatments and conditions for a certain organ or tissue [17, 21, 37]. One organ and its subordinate tissues were rarely studied together. As the two reproductive organs in the plant, the developing capsules and their seeds of *Euscaphis konishii* [57], and the developing fruits and their seeds of *Eriobotrya japonica* [49] were both collected as sample sets. The rankings of all chosen RGs were thoroughly different between its fruits and corresponding seeds, which illustrated that the RGs for two closely related organs still need to be verified experimentally. Oat endosperm is the largest component of mature oat seeds, accounting for about 90% of size [58]. The proportions of storage materials such as oil and carbohydrates are quite similar between oat seed and endosperm [6, 7]. In this study, the least stable RG of them both was *GAPDH1* (Table 3). However, the optimal RG in developing oat seeds, namely *EP*, ranked in the middle or lower under four algorithms in endosperms (Tables 2, 3). The ranking of other RGs were not similar at all when evaluated by each algorithm (Table 2). And the Ct values of all ten RGs varied in larger range in developing seeds than in developing endosperms (Fig. 4c, d). Our results strongly suggest that organs and their appurtenant tissues should be treated independently as different sample sets when they are used for RG selection.

## Conclusions

In this study, eleven candidate RGs were screened for qPCR in 18 samples of hexaploid oat, four of which were duplicated genes. With the analysis by the ΔCt method, geNorm, NormFinder and BestKeeper, our results provide a proof of concept that duplicated genes are feasible as RGs for qPCR assays in polyploid crops. The results suggested that *EIF4A* + *HNR* showed the highest stability than any other candidate RG sets across all tested samples and in developing endosperms. The combination of *EP* and *EF1A* was the best RG set for developing seeds. *UBC21*, an example of a four-copy duplicated RG, along with *HNR* was identified as the most stable RG set in shoots and roots of oat seedlings. Conversely, *GAPDH1* was regarded as the least stable in both developing seeds and endosperms. The expression pattern analysis of four various genes verified the accuracy and the reliability of optimal RG sets in developing oat seeds, endosperms and seedlings. This work is the first report for RG validation in oat and will provide useful references for future studies of gene expression based on qPCR in oat. These findings will also facilitate similar research on other closely related crops and polyploid species.

## Methods

### Plant materials

Seeds of oat cultivar Baiyan No.9 were germinated in the field at the experimental station of Northwest A&F University, Shaanxi, China (34°09′ N, 108°08′ E). Shoots and roots were segmented from two-leaf stage and three-leaf stage of seedlings, respectively. Developing oat seeds were collected at seven stages designated as stage B, C, D, E, G, I and J, based on the definitions described by Ekman et al. [7]. Corresponding endosperms of seeds from the seven stages mentioned above were separated out carefully by removing hulls, brans and embryos. Eighteen samples, including seeds, endosperms, shoots and roots of seedlings, were acquired and frozen using liquid nitrogen, then stored at -80℃ for RNA extraction.

### RNA extraction and cDNA synthesis

Total RNA of plant materials was extracted following the manufacturer’s instruction of the E.Z.N.A.® Plant RNA Kit (OMEGA) in biological triplicate. RNA quality was detected in 1% agarose gel and quantified with Nanodrop ND-2000 spectrophotometer (Thermo). cDNA synthesis was performed from 1 μg of total RNA via reverse transcription using PrimeScript™ RT reagent Kit with gDNA Eraser (Takara).

### Selection of candidate reference genes and primer design

Candidate RGs were selected from collected RGs in sequenced Gramineae species including wheat (*Triticum aestivum*), barley (*Hordeum vulgare*), sorghum (*Sorghum bicolor*), rice (*Oryza sativa*), maize (*Zea mays*) and *Brachypodium distachyon* in the internal control genes (ICG) database (https://icg.big.ac.cn/index.php/Species:Plant), and from other reported RGs in relevant articles. Corresponding sequences were retrieved by the accession numbers in NCBI (https://www.ncbi.nlm.nih.gov). A total number of nineteen candidate RGs gathered from above sources were used as query sequences to find homolog genes in oat seed transcriptome [1] by TBLASTN of NCBI local BLAST tool (blast-2.7.1 +), and subject sequences with E value no more than 1e^−30^ were chosen for BLASTX on Phytozome (https://phytozome.jgi.doe.gov) to verify their accuracy. In BLAST results, eight query RGs had no matched hits in oat seed transcriptome. Consequently, there were 11 oat RGs left and sequences of them were shown in Additional file 1.

Specific primers for qPCR were designed using Primer Premier 5.0 according to following parameters: primer length of 20–24 bp, melting temperature (T_m_) of 55–65 °C, GC content of 45–60% and product size of 80–200 bp. Detailed information is listed in Table 1. As for those candidate RGs with more than one transcript, the highly conserved part of sequences was used for primer design. Multiple alignments of duplicated genes were conducted using DNAMAN 6. All primers were synthesized by TsingKe Biotech Co., Ltd (Xi’an, China) and their products of regular PCR were verified through 1.5% agarose gels.

### Quantitative real-time PCR validation of candidate reference genes

A standard curve was generated using a series of five diluted cDNAs to calculate the amplification efficiency (E) and correlation coefficients (R^2^) of each candidate RG. The calculation of E values is as follows: E (%) = (10 − 1/slope − 1) × 100. The detailed information for all primer pairs of eleven candidate RGs is listed in Table 1.

Diluted aliquots of the reverse-transcribed cDNAs were used as templates in qPCR assays. qPCR was performed in three biological replicates with three technical replicates on QuantStudio™ 7 Flex Real-Time PCR System (Applied Biosystems, US) using ChamQ SYBR Color qPCR Master Mix (High ROX Premixed; Vazyme, Nanjing, China). Each 20 μL reaction mixture contained 10 μL 2 × qPCR Mix, 0.4 μL forward primer (10 μM), 0.4 μL reverse primer (10 μM), 1 μL cDNA (200 μg/μL) and 8.2 μL ddH2O. The qPCR program was as follows: 50 °C for 2 min and 94 °C for 30 s, 42 cycles of 94 °C for 5 s and 60 °C for 30 s. Melting curves were generated to analyze the primer specificity. To verify the stabilities of the screened RG sets, the expression patterns of *AsPKP1* (Forward primer: 5′-TCAAGAACCACATGAGCGAAAT-3′, Reverse primer: 5′-CAGACGGGCGGTAATGACTAA-3′), *AsAGPL2* (Forward primer: 5′-ATCGTCACATTCACCGCACCT-3′, Reverse primer: 5′-ATCGCCCGACAAGATCAAAATG-3′), *AsSGT1* (Forward primer: 5′-GCTGTCTTGAGGTTGGTTCTT-3′, Reverse primer: 5′-CCTGTATTTGGGCTTGCTTGG-3′) and *AsSCL14* (Forward primer: 5′-TCTGTTCTTCTATTCTGCCCTGT-3′, Reverse primer: 5′-GCTCCACCCTATCTGTACCCTCA-3′) were detected using the most and the least stable RGs, then the qPCR results were calculated by 2^−ΔΔCt^ method.

### Data analysis

The Ct values of each RG in qPCR were used to evaluate the stability using the ΔCt method, geNorm [25], NormFinder [26] and BestKeeper [27]. In geNorm analysis, the stability value (M) of each RG was generated based on the average pairwise variation (V) between all tested genes. Candidate RGs with lower M values have more stable expression. In NormFinder analysis, the stability value was evaluated by determining inter- and intra-group variations through an ANOVA-based model. The lower stability value and inter- and intra-group variation represent a more stable candidate RG. In BestKeeper analysis, the expression stability of candidate RGs was determined by the calculation of the standard deviation (SD) and coefficient of variance (CV). The lowest CV value indicates the highest stability. The geometric mean was computed to rank the stability of candidate RGs. The lower geometric mean shows the higher stability. All assays were carried out in triplicates, and the data represent the mean ± SD.

## Supplementary information

**Additional file 1.** Sequences of 11 candidate RGs.

## Data Availability

All data generated or analyzed during this study are included in this published article (and its supplementary information files).

## Abbreviations

18S: 18S Ribosomal RNA
EF1A: Elongation factor 1-Alpha
EIF4A: Eukaryotic initiation factor 4A-3
EP: Expressed protein
GAPDH1: Glyceraldehyde-3-phosphate Dehydrogenase C Subunit 1
HNR: Heterogeneous nuclear ribonucleoprotein 27C
PP2A: Protein Phosphatase 2A Subunit A3
RG: Reference gene
qPCR: Quantitative real-time polymerase chain reaction
TBC: TBC1 domain family member 22A
TUA6: Tubulin alpha-6 chain
UBC21: Ubiquitin-Conjugating Enzyme 21
UBQ10: Polyubiquitin 10
