# Supplementary material for: Evaluation of duplicated reference genes for quantitative real-time PCR analysis in genome unknown hexaploid oat (Avena sativa L.)
Source: Plant Methods. 2020 Oct 15;16:138. doi: 10.1186/s13007-020-00679-1 (PMC7560290; doi:10.1186/s13007-020-00679-1)
Supplement: Supplementary file 1 — Additional file 1. Sequences of 11 candidate RGs. [file 13007_2020_679_MOESM1_ESM.docx]

**Additional file 1:** Sequences of 11 candidate RGs

>Sequence 1 [organism= *Avena sativa*] *PP2A*, partial CDS

ATGGCTATGGTCGATGAGCCTCTGTATCCAATTGCTGTACTGATAGATGAGCTCAAAAATGAAGAAATCCCATTGCGTCTGAACTCCATCAGAAAACTTTCCACAATTGCAAGGGCACTTGGGGAAGAAAGAACCAGGAAGGAACTGATTCCTTTTCTCAGCGAAAACAATGATGATGAAGATGAGGTGCTTCTTGCAATGGCTGAAGAATTGGGTGTGTTCATTCCTTATGTTGGGGGTGTAGACCATGCTCATGTTTTGCTTCCACCGTTGGAGACATTGTCTACGGTAGAGGAAACTTGTGTCCGAGACAAAGCAGTTGAGTCGCTGTGCCGTATTGGTGCACAGATGAAGGAAAAGGACATTGTTGACTATTTCATTCCAGTAGTAAAGAGGCTAGCATCTGGTGAGTGGTTTACAGCTAGAGTATCATCCTGTGGGCTTTTCCATATAGCCTACCCAAGTGCCACTGATCCGTTGAAAACAGAACTGAGGACCATTTATGGCCAATTATGCCAAGATGACATGCCTATGGTCAGAAGGGCAGCTGCATCAAATCTTGGGAAGTTTGCTGCCACAGTTGAACAGAGCCATTTAAAGATAGAGATAATCTCAATATTTGATGATTTGACCCAAGATGATCAAGATTCAGTACGTTTATTGGCAGTTGAAGGCTGTGCCGCCCTTGGAAAATTGTTGGAACCCCAAGATTGTGTAGCACATATACTTCCAGTCATTGTCAATTTCTCCCAGGATAAATCTTGGCGTGTCCGTTATATGGTTGCCAATCAATTGTACGAGCTCTGTGAGGCTGTTGGCCCTGAGCCTACAAGAACGGACCTTGTGCCTGCATATGTTCGCCTTCTTCGTGATAATGAAGCTGAAGTACGGATTGCAGCTGCTGGAAAAGTTACTAAGTTCTGCAGGATATTAAGTCCACAACTTGCAATTCAGCACATCCTTCCATGTGTTAAGGAGTTATCATCGGATTCATCTCAGCATGTTCGCTCAGCTTTGGCTTCAGTCATTATGGGAATGGCTCCTGTTTTGGGGAAGGATGCTACCATTGAACAACTTCTTCCTATTTTTCTTTCTTTGCTCAAGGATGAATTTCCTGATGTTCGACTCAACATAATCAGCAAACTTGATCAAGTTAATCAGGTCATTGGAATTGACTTGCTGTCACAATCTCTGTTGCCGGCCATTGTAGAACTAGCAGAGGATAGGCACTGGAGGGTCCGGCTTGCAATAATTGAGTATATACCTCTGTTGGCCAGTCAGTTAGGTGTTGGTTTTTTTGATGATAAGCTGGGGGCACTTTGCATGCAATGGTTGGAGGATAAGGTGTTTTCAATAAGAGAAGCTGCCGCGAACAACTTGAAGCGTTTGGCGGAGGAGTTTGGTCCAGAGTGGGCAATGCAGCATATAATTCCTCAGGTGTTGGAGAAGAT

>Sequence 2 [organism= *Avena sativa*] *UBQ10*, complete CDS

ATGCAGATCTTTGTGAAGACCCTTACGGGCAAGACCATCACCCTCGAGGTCGAGTCTTCGGACACGATCGACAATGTCAAGGCCAAGATTCAAGACAAGGAAGGGATTCCACCGGACCAGCAGAGGCTCATCTTTGCTGGCAAGCAGCTTGAGGATGGGAGGACGCTTGCTGATTACAATATCCAGAAGGAGTCCACCCTCCACCTTGTGCTCAGGCTCAGGGGTGGCATGCAGATCTTTGTGAAGACCCTCACTGGCAAGACCATCACGCTCGAGGTCGAGTCTTCCGACACGATTGATAATGTGAAGGCAAAGATCCAGGATAAGGAGGGCATCCCCCCAGACCAGCAGCGTCTCATCTTTGCTGGCAAGCAGCTGGAGGACGGCCGCACCTTGGCTGATTATAACATCCAGAAGGAGTCGACACTTCACCTTGTCCTCAGGCTCCGCGGTGGTATGCAAATCTTTGTGAAGACCCTTACCGGCAAGACCATCACGCTCGAGGTTGAGTCCTCTGATACGATTGACAATGTCAAGGCAAAGATCCAGGACAAGGAGGGCATTCCCCCAGACCAGCAGAGGTTGATCTTTGCAGGCAAGCAGCTCGAGGATGGCCGCACTCTCGCTGATTACAACATCCAGAAGGAGTCTACCCTCCACCTGGTTCTGCGTCTCCGTGGTGGCATGCAGATCTTTGTCAAGACTTTGACTGGCAAGACCATTACCCTGGAGGTCGAGTCTTCTGACACGATTGACAATGTGAAAGCCAAGATCCAGGATAAGGAGGGCATCCCGCCAGACCAGCAGCGCCTGATCTTTGCAGGCAAGCAGCTTGAGGATGGCCGCACCCTTGCTGATTATAATATCCAGAAGGAGTCCACCCTCCATCTGGTTCTAAGGCTCAGGGGTGGCATGCAGATCTTTGTGAAGACTCTTACTGGCAAGACCATCACCCTGGAGGTGGAATCTTCAGACACCATTGACAACGTGAAGGCCAAGATCCAGGACAAAGAGGGCATTCCCCCGGACCAGCAGCGCCTGATCTTTGCTGGTAAGCAGCTCGAGGATGGCCGCACTCTGGCAGATTAA

>Sequence 3 [organism= *Avena sativa*] *EF1A*, partial CDS

ACCCGTGAGCATGCTCTCCTTGCTTTCACTCTTGGTGTCAGGCAGATGATCTGCTGCTGCAACAAGATGGATGCCACCACCCCCAAGTACTCCAAGTCTCGCTTTGAAGAAATTGTCAAGGAAGTTGGCTCCTACTTGAAGAAGGTGGGTTACAACCCTGACAAGATCCCCTTTGTTCCAATCTCTGGCTTTGAGGGCGACAACATGATTGAGAGGTCCACCAACCTTGACTGGTACAAGGGCCCCACCCTTCTTGAGGCCCTTGACCAGGTCAATGAGCCCAAGAGGCCCTCAGACAAGCCCCTCCGCCTTCCCCTTCAGGATGTCTACAAGATTGGTGGCATTGGAACTGTGCCAGTGGGGCGTGTTGAGACTGGAGTGATCAAGCCTGGCATGCTTGTCACCTTTGGCCCAACTGGGCTGACCACTGAGGTGAAGTCTGTGGAGATGCACCACGAGTCCATGCTGGAGGCTCTGCCAGGTGACAACGTTGGCTTCAATGTGAAGAACGTGGCTGTGAAGGATATCAAGCGTGGGTACGTCGCCTCCAATGCCAAGGACGACCCTGCCAAGGAGGCTGCCAACTTCGTTGCCCAGGTCATCATCATGAACCATCCTGGCCAGATTGGCAACGGCTACGCCCCAGTGCTGGACTGTCACACGTCCCACATTGCTGTCAAGTTCTCTGAAATCCAGACCAAGGTTGACAGGCGTTCTGGCAAGGAGATTGAGTCCTTCCCCAAGTTCCTCAAGAATGGTGATGCTGGCTTCGTGAAGATGATTCCCACCAAGCCCATGGTGGTGGAGACCTTCTCCCAGTACCCTCCCCTTGGCCGCTTTGCTGTGCGTGACATGAGGCAGAC

>Sequence 4 [organism= *Avena sativa*] *HNR*, complete CDS

ATGGCTTCGAAGCTTGTGGTTCTTGGAATCCCTTGGGATGTCGATACAGAAGGATTAAAAGAGTACATGGCCAAGTTTGGCCCACTGGATGATTGCATCGTTATGAAGGATCGCTCATCCGGTCGATCTCGTGGTTTTGGATACGTGACATTTTCGTCGGTAGAGGACGCAAAGAATGTCGTTAACTCCGAGCATATTCTTGGAGACCGAACTCTGGAAGTGAAGATAGCAACTCCTAAGGAAGAAATGCGAGCTCCAGGAAACAAGAAGGCTACTAGGATATTTGTTGCTCGGATTCCACAGTCTGTGGATGAGTCAATGTTTCGCAGGCATTTTGAAACTTTTGGAGAAATAATAGATCTTTATATGCCCAAGGAACTGGGTTCAAAAGACCATCGTGGAATTGGGTTTGTCACTTTCCGTAGTGCTGAATGCGTGGACAATCTTATGCAAGAAACCCACGAGATTGATGGTTCGACTGTTGTTGTTGACCGTGCAACCCCCAAGCAGGATGAAGATGCGAGACACCCTCCAAGGAGAGCAGTACAGGGCGATGGTGGTTATGGTTCATACAATGCATACATTACAGCTGCTACCAGATACGCAGCTCTGGGTGTACCAACACTGTATGATCATCCTGGACCTGCTTATGGAAGAGGATACTTAAATGAGCCCATACCAAGCAAAAAGATATTTGTTGGTAGACTTCCCCAAGAAGCAAATACTGATGATTTATGGGATTATTTTGGCCGGTTTGGCCGAATCGTGGATGCGTACATCCCAAAGGATCCTAAAAGAAGCGGACACCGTGGTTTTGGTTTTGTTACTTTTGCTGAGGATGGTGTAGCAGATCGTGTAGCACGAAGAAGTCATGAAATTCTGGGACATGAGGTTGCTGTAGACTCAGCTGCACCAGTTGAGGGTGGTGGCTCCAGAGGAGGCGGTGGTTACATGGAACCGTCAGGGCCATATGGAGCTTATGGCCCAATGATGTCTTATGGCCAGTTCTCTGGTGGTCTTGGCTATGATTATGGTTATGGTCCCAGTGGAGGTAGCGGCAGCAGCAGATCAAGAGTACATCCACGGTACAGACCATACTGA

>Sequence 5 [organism= *Avena sativa*] *EP*, complete CDS

ATGGCGATGGTGGCCCAGGCGGGCTTTGGCCTCACTCGCGTCGTCATGCTCGTCGGCGCCGGCGTGGCCGGGTCCGTCGTCCTCCGCAATGGCCGCCTCTCCGAGATCCTCACCGAGATACAGGAGTTTCTGGAGAAGGCGGACAAGGGGACGGGAGGTGGTGGTGGTGCGGACCACGGTATCAATGACGCGCTCAACGAGGTGCGCCTCCTAGCCATGCAGGTACGGCAGCTAGGTTCTCCACGTTCCATAACTGTTCTCAACGGAGGTTCTGGACAAACAGGAATGTCGGGACTGATAGTACCTGCAGCAACTGTTGGGGCACTGGGTTATGGTTATATGTGGTGGAAAGGAATCTCTTTTGCGGACCTAATGTATGTCACCAAGCAAAACATGGCAAACGTTGTTGCAAGTATGACTAAACATCTGGAGCAAGTCCAGACCTCTCTTGCTGCTGCTAAAAAGCATCTGACACAGCGCATCGAGAAGTTGGATGACAAATTGGATCAGCAAAAGGCGCTTTCTGGGCAAATAAAAGATGATGTTACTGGCGCACGATTGAAGCTTGAGACTATTGGTTCAGAAATTAAGACCATCAAGGATTTGGTCTGGGGTCTGGATGGGAAGATGGATTCAATGGAAGCTAAACAGAATTTTTCATGCGCCGGTGTGATGTACCTCTGCCAATTCATAGAGCAAAATGGTGGGAAGCTCCCGGAACGCCTGGAAGGCATTAAACCGTCTGCAAAGCGTTTTTCCGCAATTGGCATACAGGGGTTGCAACTAGCCATAGAATCGGGGGATTTCAGTGAATTCAGCAATGCTGATTCCACTGACAAGATGAGCAGGTCCAACTCGTTGAAGTCTGTCAGCTGA

>Sequence 6 [organism= *Avena sativa*] *TBC*, partial CDS

GGGGAAGGTTATGCTAACCAGAAGATCTGATCCTCTTTCACCTCCCGATTACTCCCCACGCTCTGAGAATGATCGCTATGAATATGAGAAAAATGAAGGCTCACAGGAGGTAGAAGGGCAAGCATCTGGAAACACAACCGACAACATTAGTGCAAAGAAACCAATTTCACCATCAACAAGTAGCGTGAATTCGTTACCAGATGCCCAAAGAATAGTTTCCGGGGCTAGAGCAACAGATTCTGCGAGAATTACAAAATTCACAGCTGAACTCTCTAGGCCAGCTGTTATATTAGATAAATTGCGTGAATTATCTTGGAGTGGTGTGCCCCCTTATATGCGACCTAACATATGGAGGCTACTTTTGGGATATGCACCTCCCAATGCGGATAGAAGAGAAGGTGTTCTAACGAGGAAAAGACTTGAGTATGTAGAATGTGTTTCTCAATACTACGATATTGCTGACACTGAACGCTCAGATGAAGAGATTAACATGCTTCGCCAGATTGCTGTTGACTGCCCAAGAACTGTCCCAGATGTTACCTTTTTCCAAGACCCTCAGATTCAGAAGTCTTTGGAGCGCATTTTATATACGTGGGCCATCCGGCACCCAGCAAGTGGTTATGTCCAAGGAATAAACGACCTTCTCACACCGTTCTTGATTGTGTTCTTGTCGGAGCACTTAGAAGGCAATATGGACACTTGGTCCATGGAAAAGCTTTCTTTGCAGGACGTTTCTAACATAGAAGCAGACTGCTATTGGTGTCTCTCAAAGTTCCTTGACGGCATGCAGGATCATTACACCTTTGCGCAACCAGGAATACAACGTCTTGTATTCAGGTTGAAAGAGTTGGTTCATCGGATAGATGAACCTTTATCGAAGCACATAGAGGAACAAGGATTGGAGTTCCTTCAGTTTGCCTTCCGTTGGTTCAATTGCCTTCTGATACGCGAGGTTCCTTTTCATCTCGTGACACGCCTGTGGGATACGTATCTTGCTGAAGGAGACTATTTACCAGATTTTCTCGTGTACATATCAGCGAGTTTTTTGTTAACATGGTCAGACAAGCTGCAGAAACTGGATTTCCAAGAGATGGTGATGTTCCTGCAGCACCTCCCAACCAGGAACTGGGCGCACCACGAGCTCGAGATGGTCCTCTCCAGGGCATACATGTGGCACACCATGTTCAAAAGCTCGCCCAGCCATCTCGCCAGCTAG

>Sequence 7 [organism= *Avena sativa*] *EIF4A*, partial CDS

TGTGTCGGTGGCAAGAGCATCGGCGAGGATATTAGGAAGCTCGAGGCTGGAGTGCAGGTTGTCTCTGGAACCCCAGGCAGAGTCTGTGATATGATCAAGAGGAGGACCCTGCGCACAAGGGCCATCAAGATCCTCATTCTGGACGAAGCCGACGAGATGTTGAGCAGAGGTTTTAAGGACCAGATATATGATGTCTACAGATATCTTCCCCCAGAACTTCAGGTTGTTTTGATCTCTGCAACTCTGCCCCATGACATCTTGGAGATTACTAGCAAGTTCATGACTGATCCAGTCAGGATCCTTGTGAAGCGTGATGAGTTGACCTTAGAGGGAATCAAACAATTCTTTGTTGCTGTTGAGAAGGAGGAATGGAAGTTTGATACCCTCTGTGATCTTTATGATACACTGACCATTACCCAAGCTGTCATTTTCTGCAATACTAAGAGAAAGGTGGATTGGCTTACTGAAAGAATGCGTACCAACAACTTCACTGTATCAGCTATGCATGGTGACATGCCCCAAAAGGAAAGAGATGCCATTATGAGCGAGTTCAGGGGTGGTTCAACCCGTGTTCTAATAACAACCGATGTTTGGGCCCGAGGGCTGGATGTTCAGCAGGTCTCTCTTGTCATTAACTATGATCTCCCGAATAATCGTGAGCTGTACATCCATCGCATTGGTCGCTCTGGGCGTTTTGGGCGCAAGGGTGTGGCGATCAACTTTGTGCGCAAGGACGACATCCGTATCCTGCGAGACATTGAGCAGTACTACAGCACGCAGATTGATGAGATGCCGATGAACGTGGCCGACCTGATCTGA

>Sequence 8 [organism= *Avena sativa*] *18S*, partial mRNA

TATGAAAGACGAACAACTGCGAAAGCATTTGCCAAGGATGTTTTCATTAATCAAGAACGAAAGTTGGGGGCTCGAAGACGATCAGATACCGTCCTAGTCTCAACCATAAACGATGCCGACCAGGGATCGGCGGATGTTGCTTATAGGACTCCGCCGGCACCTTATGAGAAATCAAAGTCTTTGGGTTCCGGGGGGAGTATGGTCGCAAGGCTGAAACTTAAAGGAATTGACGGAAGGGCACCACCAGGCGTGGAGCCTGCGGCTTAATTTGACTCAACACGGGGAAACTTACCAGGTCCAGACATAGCAAGGATTGACAGACTGAGAGCTCTTTCTTGATTCTATGGGTGGTGGTGCATGGCCGTTCTTAGTTGGTGGAGCGATTTGTCTGGTTAATTCCGTTAACGAACGAGACCTCAGCCTGCTAACTAGCTATGCGGAGCCATCCCTCCGCAGCTAGCTTCTTAGAGGGACTATCGCCGTTTAGGCGACGGAAGTTTGAGGCAATAACAGGTCTGTGATGCCCTTAGATGTTCTGGGCCGCACGCGCGCTACACTGATGTATTCAACGAGTATATAGCCTTGGCCGACAGGCCCGGGTAATCTTGGGAAATTTCATCGTGATGGGGATAGATCATTGCAATTGTTGGTCTTCAACGAGGAATGCCTAGTAAGCGCGAGTCATCAGCTCGCGTTGACTACGTCCCTGCCCTTTGTACACACCGCCCGTCGCTCCTACCGATTGAATGGTCCGGTGAAGTGTTCGGATCGCGGCGACGGGGGCGGTTCGCCGCCCCCGACGTCGCGAGAAGTCCATTGAACCTTATCATTTAGAGGAAGGAGAAGTCGTAACAAGGTTTCCGTAGGTGAACCTGCGGAAGGATCATTGTCGTGACCCTGACCAAAACAGACCGAGCACGCGTTATCTATTCCTGCTGAGTGGCGGCACCGTCGTCGCTCAGCCAAATCCTCGATAACCTCCTCTCCTTGGAGTTGGGGCTTGGGGTAAAAGAACCCACGGCGCCGAAGGCGTCAAGGAACACTGTGCCTAGCTTGGGGACACGACTGGCTTGCTGGCCGCTCCCCTTGCTGCAAAGCTATTTAATCCACACGACTCTCGGCAACGGATATCTCGGCTCTCGCATCGATGAAGAACGTAGCGAAATGCGATACCTGGTGTGAATTGCAGAATCCCGCGAACCATCGAGTCTTTGAACGCAAGTTGCGCCCGAGGCCATTCGGCCGAGGGCACGCCTGCCTGGGCGTCACGCTAAACACGCTCCCAACCCCTTACGGGGGAACAGGATGCGGCATTTGGCTCCCCGTCACCCAAGGGCGGTGGGCCGAAGATATGGCTGCCGGCGCATCGTGTCGGACACAGCGCGTGGTGAGCGTCCTCGCTATACTTACCGCAGTGTCTCCGACACATAGCCGGCGTGATGGCCTAGAATGACCCTTGTAACGGTGCGCATGACGCTCCGACCGCGACCCCAGGTCAGGCGGGACTACCCGCTGAATTTAAGCATATAAATAAGCGGAGGAGAAGAAACTTACAAGGATTCCCCTAGTAACGGCGAGCGAACCGGGAACAGCCCAGCTTGAGAATCGGGCGGCCCTGTCGTTCGAATTGTAGTCTGGAGAGGCGTCCTCAGCGACGGACCGAGCCCAAGTCCCCTGGAAAGGGGCGCCTGGGAGGGTGAGAGCCCCGTCCGGCCCGGACCCTGTCGCATCACGAGGCGCCGTCAACGAGTCGGGTTGTTTGGGAATGCAGCCCAAATCGGGCGGTAGACTCCGTCCAAGGCTAAATACAGGCGAGAGACCGATAGCGAACAAGTACCGCGAGGGAAAGATGAAAAGGACTTTGA

TCAAAGTCCTTTTCATCTTTCCCTCGCGGTACTTGTTCGCTATCGGTCTCTCGCCTGTATTTAGCCTTGGACGGAGTCTACCGCCCGATTTGGGCTGCATTCCCAAACAACCCGACTCGTTGACGGCGCCTCGTGATGCGACAGGGTCCGGGCTGGACGGGGCTCTCACCCTCCCAGGCGCCCCTTTCCAGGGGACTTGGGCTCGGTCCGTCGCTGAGGACGCCTCTCCAGACTACAATTCGAACGACAGGGCCGCCCGATTCTCAAGCTGGGCTGTTCCCGGTTCGCTCGCCGTTACTAGGGGAATCCTTGTAAGTTTCTTCTCCTCCGCTTATTTATATGCTTAAATTCAGCGGGTAGTCCCGCCTGACCTGGGGTCGCGGTCGGAGCGTCATGCGCACCGTTACAAGGGTCATTCTAGGCCATCACGCCGGCTATGTGTCGGAGACACTGCGGTAAGTATAGCGAGGACGCTCACCACGCGCTGTGTCCGACACGATGCGCCGGCAGCCATATCTTCGGCCCACCGCCCTTGGGTGACGGGGAGCCAAATGCCGCATCCTGTTCCCCCGTAAGGGGTTGGGAGCGTGTTTAGCGTGACGCCCAGGCAGGCGTGCCCTCGGCCGAATGGCCTCGGGCGCAACTTGCGTTCAAAGACTCGATGGTTCGCGGGATTCTGCAATTCACACCAGGTATCGCATTTCGCTACGTTCTTCATCGATGCGAGAGCCGAGATATCCGTTGCCGAGAGTCGTGTGGATTAAATAGCTTTGCAGCAAGGGGAGCGGCCAGCAAGCCAGTCGTGTCCCCAAGCTAGGCACAGTGTTCCTTGACGCCTTCGGCGCCGTGGGTTCTTTTACCCCAAGCCCCAACTCCAAGGAGAGGAGGTTATCGAGGATTTGGCTGAGCGACGACGGTGCCGCCACTCAGCAGGAATAGATAACGCGTGCTCGGTCTGTTTTGGTCAGGGTCACGACAATGATCCTTCCGCAGGTTCACCTACGGAAACCTTGTTACGACTTCTCCTTCCTCTAAATGATAAGGTTCAATGGACTTCTCGCGACGTCGGGGGCGGCGAACCGCCCCCGTCGCCGCGATCCGAACACTTCACCGGACCATTCAATCGGTAGGAGCGACGGGCGGTGTGTACAAAGGGGAGGGACGTAGTCAACGCGAGCTGATGACTCGCGCTTACTAGGCATTCCTCGTTGAAGACCAATAATTGCAATGATCTATCCCCATCACGATGAAATTTCCCAAGATTACCCGGGCCTGTCGGCCAAGGCTATATACTCGTTGAATACATCAGTGTAGCGCGCGTGCGGCCCAGAACATCTAAGGGCATCACAGACCTGTTATTGCCTCAAACTTCCGTCGCCTAAACGGCGATAGTCCCTCTAAGAAGCTAGCTGCGGAGGGATGGCTCCGCATAGCTAGTTAGCAGGCTGAGGTCTCGTTCGTTAACGGAATTAACCAGACAAATCGCTCCACCAACTAAGAACGGCCATGCACCACCACCCATAGAATCAAGAAAGAGCTCTCAGTCTGTCAATCCTTGCTATGTCTGGACCTGGTAAGTTTCCCCGTGTTGAGTCAAATTAAGCCGCAGGCTCCACGCCTGGTGGTGCCCTTCCGTCAATTCCTTTAAGTTTCAGCCTTGCGACCATACTCCCCCCGGAACCCAAAGACTTTGATTTCTCATAAGGTGCCGGCGGAGTCCTATAAGCAACATCCGCCGATCCCTGGTCGGCATCGTTTATGGTTGAGACTAGGACGGTATCTGATCGTCTTCGAGCCCCCAACTTTCGTTCTTGATTAATGAAAACATCCTTGGCAAATGCTTTCGCAGTTGTTCGTCTTTCATA

>Sequence 9 [organism= *Avena sativa*] *GAPDH1*, partial CDS

CGTCGCCGTCAACGACCCCTTCATCACCACCGAGTACATGACCTACATGTTCAAGTACGACACCGTGCACGGCCACTGGAAGCACAGCGACATCAAGCTCAAGAACGACAAGACTCTCCTCTTCGGCGAGAAGGCGGTTACTGTCTTTGGCGTCAGGAACCCTGAAGAAATCCCATGGGCTGAGGCTGGTGCCGACTATGTCGTTGAGTCCACCGGTGTATTCACTGACAAGGACAAGGCTGCTGCTCACTTGAAGGGTGGTGCCAAGAAGGTGGTCATTTCAGCCCCTAGCAAAGATGCCCCTATGTTTGTGGTTGGTGTCAACGAGGACAAGTACACTTCTGATGTTAACATTGTCTCAAACGCTAGCTGCACAACAAACTGTCTTGCTCCCCTTGCCAAGATCATTAATGACAACTTCGGTATTGTTGAGGGTCTGATGACCACTGTTCATGCCATCACTGCCACTCAGAAGACTGTTGATGGTCCCTCAAGCAAGGACTGGAGAGGTGGGAGAGCTGCTAGCTTCAACATCATTCCCAGCAGCACTGGCGCTGCCAAGGCTGTTGGTAAGGTTCTTCCTGTGTTGAACGGCAAGCTTACCGGTATGTCATTCCGGGTACCCACCGTGGATGTGTCAGTTGTTGATCTCACAGTCAGAATTGAGAAGGCTGCATCATACGAGGACATCAAAAAGGCTATCAAGGCTGCATCTGAGGGAAACCTCAAGGGAATCATGGGCTATGTTGAGGAAGATTTGGTTTCCACCGACTTCATTGGCGACAGCAGGTCGAGCATCTTTGACGCCAAGGCTGGAATTGCTCTTAACGACAACTTCGTCAAGCTTATTTCGTGGTACGACAACGAGTGGGGTTACAGCAACCGTGTTGTCGACCTGATCCGCCACATGGCCAAGACTCAGTAG

CGGTGTTTTCACTGACAAGGACAAGGCTGCAGCTCACATCAAGGGTGGTGCCAAGAAGGTCATCATTTCTGCTCCCAGCAAGGACGCTCCCATGTTCGTCATGGGTGTCAACGAGAAGGAGTACACCTCTGACATCACTATTGTCTCCAACGCTAGCTGCACCACTAACTGCCTTGCTCCCCTTGCTAAGGTTATCAATGACAAGTTTGGCATTGTTGAGGGTTTGATGACCACTGTTCACGCCATGACTGCAACCCAGAAGACTGTTGATGGTCCCTCAAGCAAGGACTGGAGAGGTGGAAGGGCTGCTAGCTTCAACATCATTCCCAGCAGCACTGGAGCTGCAAAGGCTGTTGGCAAGGTGCTCCCTGTCCTCAACGGAAAGTTGACAGGAATGGCCTTCCGTGTTCCAACTGTTGATGTTTCTGTTGTTGACCTGACCGTTAGACTTGAGAAGGCAGCCACCTATGAGCAGATCAAGGCTGCAATCAAGGAGGAGTCCGAGGGTAAGCTCAAGGGCATTCTGGGTTATGTTGATGAGGACCTTGTTTCCACTGACTTCCAGGGTGACAACAGGTCCAGCATCTTTGACGCCAAGGCTGGGATTGCTCTGAACGACAACTTTGTCAAGCTTGTGTCCTGGTACGACAACGAGTGGGGCTACAGCACCCGTGTGGTCGACCTGATCCGTCACATCCACGGCACCAAGTGA

>Sequence 10 [organism= *Avena sativa*] *TUA6*, complete CDS

ATGAGGGAGATCCTGCACATCCAGGGCGGGCAGTGCGGAAACCAGATCGGCTCCAAGTTCTGGGAGGTGGTCTGCGACGAGCACGGCATCGACCCCACTGGCCGCTACGTCGGCACCGCCGATCTCCAGCTCGAGCGCGTCAACGTCTACTACAATGAAGCCTCCTGCGGCCGCTTTGTGCCCCGCGCCGTGCTCATGGACCTTGAGCCCGGCACCATGGACAGCGTCCGCACCGGGCCCTATGGCCAGATCTTCCGCCCTGACAATTTCGTCTTCGGCCAGTCCGGCGCCGGCAACAACTGGGCCAAGGGGCACTACACCGAGGGAGCTGAGCTCATCGACTCGGTAATGGATGTCGTGAGGAAGGAGGCGGAGAACTGCGACTGCCTCCAAGGTTTTCAGGTTTGCCATTCCCTCGGAGGAGGCACTGGGTCTGGAATGGGCACACTCTTGATCTCCAAGATCAGGGAGGAGTACCCTGACAGGATGATGCTCACATTTTCTGTCTTCCCTTCACCAAAAGTCTCAGACACAGTTGTTGAACCCTACAATGCTACACTCTCTGTCCATCAGTTGGTTGAGAACGCCGATGAGTGTATGGTTCTTGACAATGAGGCGCTTTACGACATCTGCTTCCGTACCCTGAAGCTGACGACTCCCAGCTTTGGAGACCTTAACCACTTGATCAGTGCTACAATGAGCGGTGTCACATGCTGCCTTCGCTTCCCAGGCCAGCTGAACTCTGATCTCCGGAAGCTTGCAGTCAACTTGATCCCGTTCCCAAGGCTTCACTTTTTCATGGTTGGCTTTGCGCCACTCACCTCCCGTGGCTCCCAGATGTACCGTGCCCTCACCGTTCCTGAGCTCACTCAGCAAATGTGGGACTCAAAGAACATGATGTGCGCTGCTGACCCGCGCCACGGTCGCTACCTGACAGCCTCTGCCATGTTCCGTGGGAAGATGAGCACCAAGGAGGTTGACGAGCAGATGATCAATGTGCAGAACAAGAACTCGTCATACTTTGTGGAGTGGATCCCGCACAACGTCAAGTCCAGCGTCTGCGACATCCCTCCGCGTGGCCTCTCCATGTCTTCCACCTTTGTTGGCAACTCGACCTCCATCCAGGAGATGTTCTGCCGTGTGAGCGAGCAGTTCACTGCTATGTTCAGGAGGAAGGCTTTCTTGCATTGGTACACGGGTGAGGGCATGGACGAGATGGAGTTCACCGAAGCTGAGAGCAACATGAATGACCTGGTCTCCGAGTACCAGCAGTATCAGGACGCGACAGCCGACGAGGAGGGTGAGTATGAGGACGAGGAGGAGGCGATCCAGGACGAGTGA

ATGACTTTCCTTCTGTTACGTTCAGTCCTTGCTTTACCCATCCTTGTTGCAATGCCTGAAATTAATTCATTTATGTGCTTCTCTTCTGTTTATAATAGGGCGAAGATGAGGGAAATCCTGCACATCCAGGGTGGGCAGTGTGGCAACCAGATCGGTGCCAAGTTCTGGGAGGTGGTGTGTGACGAGCACGGCATTGACCCAACTGGGCGGTATGTCGGCACTTCTGACCTGCAGTTGGAGCGTGTCAATGTCTACTACAACGAGGCCTCATGTGGGCGCTTTGTGCCCCGTGCTGTGCTCATGGACCTTGAGCCCGGTACCATGGACAGTGTCCGCACTGGACCCTATGGGCAGATCTTCCGCCCTGACAACTTTGTCTTTGGGCAATCTGGTGCGGGTAACAATTGGGCCAAGGGTCACTACACCGAGGGTGCTGAGCTTATCGACTCTGTTTTGGATGTTGTGAGGAAGGAGGCTGAGAACTGTGACTGCCTGCAAGGATTCCAAGTATGCCACTCTCTTGGTGGTGGTACTGGATCTGGCATGGGTACCCTGTTGATATCCAAGATCAGGGAAGAGTACCCTGACCGCATGATGCTCACATTCTCAGTGTTCCCCTCACCAAAGGTGTCCGATACTGTTGTTGAGCCATACAATGCCACTCTCTCAGTCCATCAGTTGGTTGAGAATGCTGATGAGTGCATGGTTCTTGACAACGAGGCTCTGTATGACATCTGTTTCAGGACTCTCAAGCTGACAACGCCTAGCTTTGGGGATTTGAACCATCTAATCTCTGCTACCATGAGTGGAGTCACCTGTTGCCTAAGGTTCCCTGGACAGCTGAATTCCGACCTCCGGAAGCTGGCAGTGAACCTTATCCCCTTCCCTCGTCTCCACTTCTTCATGGTTGGCTTTGCTCCCCTGACATCCCGTGGATCTCAGCAGTACCGTGCCCTCACAGTCCCTGAGCTCACACAGCAAATGTGGGACTCCAAGAACATGATGTGCGCCGCCGATCCTCGCCATGGCCGTTACCTCACTGCCTCTGCCATGTTCCGTGGAAAGATGAGCACAAAGGAGGTTGATGAGCAGATGATCAACGTTCAGAACAAGAACTCTTCCTACTTTGTGGAGTGGATCCCCAACAATGTGAAGTCCAGCGTCTGTGACATCCCACCTACGGGTCTCTCAATGGCGTCCACCTTCATTGGCAACTCAACCTCCATCCAGGAGATGTTCCGGAGGGTGAGTGAGCAGTTCACTGCCATGTTCAGGAGGAAGGCTTTCTTGCATTGGTACACCGGTG

TGCCTTCAAGGTTTCCAAGTATGCCACTCTCTTGGTGGTGGTACTGGATCTGGCATGGGGACACTTTTGATATCCAAGATCAGGGAGGAGTACCCAGACCGCATGATGCTAACATTCTCTGTTTTCCCCTCACCGAAAGTATCTGATACTGTGGTTGAGCCGTACAATGCCACTCTTTCTGTCCATCAGTTGGTTGAGAATGCAGATGAGTGCATGGTTCTTGACAATGAGGCTCTTTATGACATCTGTTTCAGGACTCTCAAGCTAACCACTCCCAGCTTTGGAGATCTGAACCATTTGATCTCTGCCACCATGAGCGGAGTAACATGCTGCTTGAGGTTCCCTGGTCAGCTGAACTCCGACCTCCGGAAGTTGGCAGTGAACCTTATCCCCTTCCCACGTCTCCACTTCTTCATGGTCGGGTTCGCCCCCTTGACCTCGCGCGGCTCCCAGATGTACCGCTCCCTCACGGTCCCCGAGCTGACCCAGCAGATGTGGGACTCCAAGAACATGATGTGCGCGGCGGACCCCCGCCACGGCCGCTACCTGACTGCCTCGGCCATGTTCCGCGGCAAGATGAGCACCAAGGAGGTGGACGAGCAGATGATCAACGTGCAGAACAAGAACTCGTCCTACTTCGTGGAGTGGATCCCCAACAACGTGAAGTCGAGCGTGTGCGACATCGCGCCGCGGGGCCTGTCGATGGCGTCGACCTTCGTGGGCAACTCGACGTCGATCCAGGAGATGTTCAGGCGTGTGAGCGAGCAGTTCACGGCCATGTTCAGGCGGAAGGCCTTCCTGCACTGGTACACGGGCGAGGGCATGGACGAGATGGAGTTCACCGAGGCTGAGAGCAACATGAACGACCTCGTCGCGGAGTACCAGCAGTACCAGGACGCGACCGCCGACGAGGAGGGCGACTACGAGGACGAGGAGGACCTGCAGGCCGACGAGGAGTGA

>Sequence 11 [organism= *Avena sativa*] *UBC21*, complete CDS

ATGCAGGCATCTAGAGCTAGGCTCTTCAAGGAGTACAAGGAGGTGCAGCGAGAAAAGTCAGCGGACCCAGATATCCAGTTAATCTGTGATGATTCAAATATATTCAAGTGGACCGCTCTAATTAAGGGCCCATCGGAGACACCTTTTGAAGGTGGTGTATTTCAGCTTGCATTCTCAATTCCTGAGCAGTATCCTCTGCTGCCTCCACAAGTCCGCTTCATGACCAAAATTTTCCACCCAAATGTTCACTTCAAGACAGGTGAAATATGCCTGGATATATTGAAGAATGCTTGGAGCCCTGCATGGACCCTACAATCTGTCTGCAGAGCCATAATTGCTTTGATGGCCCATCCAGAAGCAGATAGCCCCCTTAACTGTGACTCAGGCAATCTCCTGCGCTCAGGCGATATCAGAGGCTACCAATCTATGGCACGAATGTATACTAGGCTAGCTGCCATGCCAAAGAAAGATTAG

ATGCAGGCATCTAGAGCTAGGCTCTTCAAGGAGTACAAGGAGGTGCAGCGAGAAAAGTCAGCGGACCCAGATATCCAGTTAATCTGTGATGATTCAAATATATTCAAGTGGACCTCTGTAATTAAGGGCCCATCGGAGACACCTTTTGAAGGTGGTGTATTTCAGCTCGCATTCTCAATTCCTGAGCAGTATCCTCTGCTGCCTCCACAAGTCCGCTTCATGACCAAAATTTTCCACCCAAATGTTCACTTCAAGACAGGTGAAATATGCCTGGATATATTGAAGAATGCTTGGAGCCCTGCATGGACCCTACAATCTGTCTGCAGAGCCATAATTGCTTTGATGGCCCATCCAGAAGCAGATAGCCCCCTTAACTGTGACTCAGGCAATCTCCTGCGCTCAGGCGATATCAGAGGCTACCAATCTATGGCACGAATGTATACTAGGCTAGCTGCCATGCCAAAGAAAGATTAG

ATGCAGGCATCTAGAGCTAGGCTTTTCAAGGAGTACAAGGAGGTGCAGCGAGAAAAGTCAGCCGACCCTGATATCCAATTAATCTGTGATGATTCGAATATATTCAAGTGGACTGCTCTAATCAAGGGCCCATCGGAGACACCTTTTGAAGGTGGTGTATTTCAACTTGCATTCTCAATTCCCGAGCAGTATCCTCTACTGCCTCCACAAGTCCGCTTCCTGACCAAAATTTTCCACCCAAATGTTCACTTCAAGACAGGTGAAATATGCCTGGATATATTGAAGAATGCGTGGAGCCCTGCATGGACCCTACAATCTGTCTGCAGAGCCATTATTGCGTTGATGGCCCATCCAGAAGCAGATAGCCCACTTAACTGTGACTCAGGCAATCTCCTGCGCTCAGGTGATATCAGAGGCTACCAGTCCATGGCGCGGATGTATACTAGGTTAGCTGCCATGCCAAAGAAAGAGTAA

ATGCAGGCATCTAGAGCTAGGCTTTTCAAGGAGTACAAGGAAGTGCAGCGAGAAAAGTCAGCCGACCCTGATATCCAATTAATCTGTGATGATTCGAATATATTCAAGTGGACTGCTCTAATCAAGGGCCCATCGGAGACACCTTTTGAAGGTGGTGTATTTCAACTTGCATTCTCAATTCCCGAGCAGTATCCTCTACTGCCTCCACAAGTCCGCTTCCTGACCAAAATTTTCCACCCAAATGTTCACTTCAAGACAGGTGAAATATGCCTGGATATATTGAAGAATGCGTGGAGCCCTGCATGGACCCTACAATCTGTCTGCAGAGCCATTATTGCGTTGATGGCCCATCCAGAAGCAGATAGCCCACTTAACTGTGACTCAGGCAATCTCCTGCGCTCAGGTGATATCAGAGGCTATCAGTCCATGGCGCGGATGTATACTAGGTTAGCTGCCATGCCAAAGAAAGAGTAA
